# Consanguineous marriages and their association with women’s reproductive health and fertility behavior in Pakistan: secondary data analysis from Demographic and Health Surveys, 1990–2018

**DOI:** 10.1186/s12905-022-01704-2

**Published:** 2022-04-14

**Authors:** Sarosh Iqbal, Rubeena Zakar, Florian Fischer, Muhammad Zakria Zakar

**Affiliations:** 1 Institute of Social and Cultural Studies, University of the Punjab, Lahore, Pakistan; 2 Department of Public Health, Institute of Social and Cultural Studies, University of the Punjab, Lahore, Pakistan; 3 Institute of Public Health, Charité – Universitätsmedizin Berlin, Charitéplatz 1, 10117 Berlin, Germany; 4 Institute of Gerontological Health Services and Nursing Research, Ravensburg-Weingarten University of Applied Sciences, Weingarten, Germany; 5 University of Okara, Okara, Pakistan

**Keywords:** Consanguinity, Consanguineous union, Reproduction, Sexual and reproductive health

## Abstract

**Background:**

Pakistan has been showing consistently the highest prevalence of consanguinity. The popularity of consanguineous marriages is not declining in the country, because of social, cultural, and religious beliefs as well as economic advantages. However, couples also face various health-related implications, such as poor pregnancy outcomes or multiple reproductive and fertility consequences, having adverse effects on mothers and their children. This research investigated the trend of consanguineous marriages and their association with women’s reproductive health and fertility behavior in Pakistan from 1990 to 2018.

**Methods:**

This study is based on secondary data analysis, using all four waves of the Pakistan Demographic Health Surveys carried out from 1990 to 2018. The analysis is limited to women aged 15–49 years, who had given birth in the previous five years preceding each survey. Sampling weights were calculated and subsequently weighted analysis was conducted. Descriptive statistics, bivariable and multivariable logistic regression analysis were performed to determine the association of consanguinity with multiple characteristics related to socio-demographics, co-variates, and women’s reproductive health and fertility behaviors.

**Results:**

The findings revealed a high but overall stable trend of consanguinity prevalence of about 63% during the last three decades. Consanguineous marriages were more prevalent amongst young and uneducated women, living in rural areas, with poorer wealth status and having less exposure to mass media to access information. A strong association of consanguinity was observed with women’s reproductive health and fertility behavior, particularly for women who gave first birth at a younger age, had multi-gravida pregnancies, multi-parity, pregnancy termination, ANC visits, and higher fertility.

**Conclusion:**

Consanguineous marriages are predominant in the patriarchal society of Pakistan. Findings revealed that consanguinity contributes significantly to women’s reproductive health and fertility behaviors. Appropriate counseling, educational, and health promotional programs related to consanguinity should be designed and launched at the community level to raise awareness about risks towards women’s reproductive health and fertility.

## Background

Consanguinity is termed as wedlock or marriage between close blood relations or biological kin. Consanguineous marriages have been very common since the early existence of humanity. According to a rough estimate, nearly one billion (20%) of the global population live in communities with a preference for consanguineous marriages [1, 2], predominantly in Muslim countries of the Middle East, Africa, and South Asia [3, 4]. With 65%, Pakistan has one of the highest rates of cousin marriages globally, followed by India (55%), Saudi Arabia (50%), Afghanistan (40%), Iran (30%), Egypt, and Turkey (20%) [5]. The prevalence of consanguineous unions differs amongst countries due to socio-demographic factors, such as geography (urban–rural residential community, isolated area, and population), religion, education, socio-economic status, a familial pattern towards early marriages, or consanguinity between parents [4, 6–12]. Although the incidence of consanguineous unions somehow decreased with urbanization, modernization, and smaller/nuclear families, however, it is still in practice [13].

Pakistan, a multi-cultural country with diverse caste systems, has been shown consistently the highest prevalence of consanguinity [14, 15]. Consanguineous marriages are encouraged in the country due to multiple reasons, e.g. to strengthen interfamily ties between close family members, a preference to the same caste and status, fear of incompatibility or difficulty in finding the right partner outside the family, security of being familiar with spouse and in-laws before marriage, restriction for socialization with the opposite gender, and financial constraints especially for dowry [16]. However, various socio-cultural and health-related implications have been identified for consanguineous couples [5]. Owing to shared alleles, consanguinity may lead to genetic disorders [17, 18], poor pregnancy outcomes, or multiple reproductive and fertility consequences, having adverse effects on mothers, their children, family, and society as a whole [5, 13]. A strong association of consanguineous marriages has been reported with increased rates of abortion, stillbirths, pregnancy terminations, low birth weights, increased mortality, and congenital malformations [13, 17, 19–23]. Furthermore, a low preference for contraception, extended childbearing age, and higher fertility has also been observed in such unions [8]. Although consanguineous marriages are also linked with poor pregnancy outcomes [22] and higher reproductive risks in Pakistan, nonetheless these are also associated with increased fertility rates and larger family size [24, 25].

Consanguineous unions have remained under continuous investigation by social scientists, medical researchers, biologists, and physicians. However, it received less attention in mainstream demographic research. Although multiple studies are available regarding the effects of consanguineous marriages on either reproductive health or fertility behaviors [22, 24, 25], nevertheless, there is a need to examine and explain the trends of consanguineous marriages and their association with women’s reproductive health and fertility behavior in Pakistan. To our best knowledge, this is the first paper to examine trends in consanguineous marriages in Pakistan in recent decades. Therefore, this research is an attempt to investigate the differentials in reproductive health and fertility behaviors over almost three decades (1990–2018).

## Methods

### Study design and data source

This research performed secondary data analysis, using all four waves of Pakistan Demographic and Health Surveys (PDHSs), carried out during 1990–1991 (wave 1) [26], 2006–07 (wave 2) [27], 2012–2013 (wave 3) [28], and 2017–2018 (wave 4) [29]. These PDHSs are characterized as nationally representative and large-scale cross-sectional household surveys, conducted under the international series of MEASURE Demographic and Health Survey (DHS) Program and funded by the United States Agency for International Development (USAID). These surveys were carried out by the National Institute of Population Studies (NIPS) with the technical assistance of ICF International and the Pakistan Bureau of Statistics. A series of PDHSs is the largest household and publicly available dataset, with information on women’s reproductive health, fertility behavior, marital status, and other socio-demographic variables. Each PDHS applied a random two-stage cluster sampling design, wherein firstly rural and urban sampling units were chosen, followed by the selection of eligible households with ever-married women (aged 15–49 years) [26–29].

Within each wave of PDHS, varied numbers of field teams collected data, each comprised of one male and three female interviewers, field editor, and supervisor. All teams were closely monitored by quality controllers, provincial/regional field coordinators, as well as the NIPS and ICF core team. Simultaneously, data editing, processing, and double data entry of completed questionnaires were also completed. Each wave of PDHSs used various questionnaires at household, community, women, and men levels for data collection. This research used the standard female questionnaire for data analysis, administered to women (aged 15–49 years), using the face-to-face method [26–29], and including questions about consanguinity, women’s reproductive health, and fertility behaviors. The response rate was recorded between 93 and 94.5% for each wave of PDHS [26–29].

This research limited the analysis to the women of reproductive age (15–49 years), who had given birth in the previous years preceding each of the four PDHS waves from 1990 to 2018. Therefore, the sample size used for this study was 4,061, 5,677, 7,446, and 6,711, for 1990–1991, 2006–2007, 2012–2013, and 2017–2018, respectively.

### Variables

*Outcome variable*: The outcome variable for this research is the marital status of respondents, i.e. non-consanguineous marriage versus consanguineous marriage. Further, types of consanguineous marriages were divided into three categories, including married to paternal first cousins, married to maternal first cousins, and married to relatives other than first paternal or maternal cousins.

*Reproductive health and fertility behavior*: Various variables related to women’s reproductive health and fertility behavior were selected based on literature [1, 5, 8, 13, 19, 21, 22, 25]. These included age at first birth (< 20 years, 20–34 years, 35–49 years), gravidity (1–2, 3–5, 6 and above), parity (1–2, 3–4, 5 children, and above), number of living children (none, 1–2, 3–4, 5 and above), ever terminated pregnancy (yes/no), antenatal care (ANC) visits during last pregnancy (less than 4 visits, 4 visits and above), skilled birth attendants (SBA) at delivery during the last pregnancy (yes/no), ideal family size (1–2, 3–4, 5 and above), fertility intention/desired family size (wants more children, wants no more, i.e. undecided, sterilized, declared infecund), and current use of contraception (yes/no).

*Sociodemographic characteristics and co-variates*: The sociodemographic characteristics included geographical classification (urban, rural), regions/provinces (Islamabad, Sindh, Punjab, Khyber Pakhtunkhwa, FATA, Baluchistan, Gilgit Baltistan), respondent’s age in years (15–24, 25–34, 35 years and above), education status (uneducated, primary, secondary, higher) as well as employment status of respondents and their husbands (unemployed, employed), and wealth quintile (richest, richer, middle, poorer, poorest). Other co-variates included exposure to mass media to access information (yes/no) and respondents’ healthcare decision-making autonomy (yes/no).

### Statistical analysis

Data analysis was conducted using SPSS version 21. Initially, sample weights were calculated and weighted analysis was performed. Descriptive statistics were presented in the form of frequencies and percentages. For bivariate analyses, cross-tabulation and chi-square tests were applied. Afterward, bivariable and multivariable logistic regression analyses were performed to measure the association of consanguineous marriages with sociodemographic characteristics, as well as women’s reproductive health and fertility behaviors. During regression analysis, odds ratios (OR) and adjusted odds ratios (AOR) were calculated with a 95% confidence interval (CI). A *p*-value ≤ 0.05 was considered statistically significant.

### Ethical considerations

Procedures and questionnaires for all DHS surveys have been reviewed and approved by ICF Institutional Review Board. Before each interview, an informed consent statement was read to the respondent, who had the chance to accept or decline to participate. The informed consent statements emphasized that participation is voluntary and provided details regarding the purpose of the interview, the expected duration of the interview, interview procedures, potential risks and potential benefits to respondents, as well as the contact information who could provide the respondent with more information about the interview.

## Results

### Sample characteristics

Table 1 highlights respondents’ socio-demographic characteristics and other co-variates for the four PDHS waves from 1990 to 2018. The majority of women were of 25–34 years of age, uneducated (but with a lower trend over time: 79.2%, 64.6%, 55.8%, and 47.9%), and resided in rural areas (70.9%, 69.8%, 59.9%, and 66.5%). About three quarter or even more of the women were unemployed. Contrary to respondents, most of their husbands acquired a secondary level of education and almost all of them were employed. Although, findings revealed that about 60–70% of women had exposure to mass media, about half of the participants had no autonomy in healthcare decision-making.

**Table 1:** Sociodemographic characteristics and co-variates of respondents

Characteristics	PDHS (1990–1991)		PDHS (2006–2007)		PDHS (2012–2013)		PDHS (2017–2018)	
	n = 4061		n = 5677		n = 7446		n = 6711	
	f	%	f	%	f	%	f	%
**Sociodemographic characteristics**								
*Regions/Provinces*								
Punjab	2441	60.1	3182	56.1	4180	56.1	3453	51.5
Sindh	894	22	1404	24.7	1714	23.0	1571	23.4
Baluchistan	159	3.9	264	4.6	348	15.0	377	5.6
Khyber Pakhtunkhwa ^a^	567	14	827	14.6	1117	4.7	1101	16.4
Gilgit Baltistan*	–	–	–	–	56	0.7	–	–
Islamabad*	–	–	–	–	31	0.4	54	0.8
FATA*	–	–	–	–	–	–	156	2.3
*Geographical classification*								
Urban	1184	29.1	1714	30.2	2244	30.1	2248	33.5
Rural	2877	70.9	3962	69.8	5202	69.9	4463	66.5
*Respondents’ age*								
15–24 years	983	24.2	1334	23.5	1748	23.5	1545	23.0
25–34 years	2061	50.7	2952	52.0	4038	54.2	3725	55.5
35 years and above	1017	25	1390	24.5	1659	22.3	1440	21.5
*Respondents’ education status*								
Uneducated	3214	79.2	3668	64.6	4155	55.8	3212	47.9
Primary	373	9.2	854	15.0	1230	16.5	1097	16.3
Secondary	427	10.5	813	14.3	1380	18.5	1492	22.2
Higher	47	1.2	341	6.0	682	9.2	911	13.6
*Husbands’ education status*								
Uneducated	1946	48.2	2007	35.4	2451	33.0	1889	28.7
Primary	698	17.3	935	16.5	1211	16.3	1085	16.5
Secondary	1213	30.0	1904	33.5	2547	34.3	2316	35.2
Higher	181	4.5	812	14.3	1216	16.4	1293	19.6
*Respondents’ employment status*								
Unemployed	3389	83.5	4026	71.0	5378	72.2	5528	82.4
Employed	669	16.5	1647	29.0	2068	27.8	1180	17.6
*Husbands’ employment status*								
Unemployed	77	2.0	174	3.1	123	1.7	173	2.6
Employed	3844	98.0	5501	96.9	7322	98.3	6415	97.4
*Wealth quintile*								
Richest	1085	26.7	1029	18.1	1272	17.1	1248	18.6
Richer	923	22.7	1066	18.8	1469	19.7	1349	20.1
Middle	755	18.6	1099	19.4	1464	19.7	1371	20.4
Poorer	689	17.0	1194	21.0	1544	20.7	1299	19.4
Poorest	609	15.0	1289	22.7	1698	22.8	1444	21.5
**Covariates**								
Mass media exposure*^b^								
Yes	1636	59.5	–	–	5241	70.6	4254	63.4
No	2404	40.5	–	–	2184	29.4	2454	36.6
Respondents’ healthcare decision-making autonomy*								
Yes	–	–	–	–	3511	47.9	3054	46.2
No	–	–	–	–	3826	52.1	3550	53.8

^a^Khyber Pakhtunkhwa was formerly known as North-West Frontier Province (NWFP), as previously reported in PDHS 1990–1991 and 2006–2007

^b^Mass media exposure refers to the frequency of reading a newspaper or watching TV or listening to radio

^*^Missing information indicates the non-availability of data within the respective PDHS wave

### Trend of consanguineous marriages

Table 2 shows the proportion of consanguineous marriages amongst women of reproductive age from 1990 to 2018. Results indicate that about two-thirds of women were married to their cousins, more frequently with paternal first cousins than maternal ones. Findings revealed an overall stable proportion of consanguinity prevalence during the last three decades. However, even a slightly upward trend of consanguineous marriages was witnessed from 63.0% in 1990–1991 to 67.9% in 2007–2008, followed by a gradual downwards trend. Similarly, the pattern of marriages with paternal and maternal first cousins decreased during the period from 1990 to 2013, nonetheless, slightly increased in 2017–2018.

**Table 2:** Measures to describe consanguinity, reproductive health and fertility behavior

Characteristics	PDHS (1990–1991)		PDHS (2006–2007)		PDHS (2012–2013)		PDHS (2017–2018)	
	n = 4061		n = 5677		n = 7446		n = 6711	
	f	%	f	%	f	%	f	%
**Consanguinity**								
*Marital status*								
Non-consanguineous marriages	1500	37.0	1819	32.1	2519	33.8	2440	36.4
Consanguineous marriages	2550	63.0	3855	67.9	4926	66.2	4270	63.6
*Type of consanguineous marriages*								
Married to paternal first cousins	1223	47.9	1814	47.1	2061	41.9	1882	44.1
Married to maternal first cousins	856	33.6	1198	31.1	1558	31.7	1433	33.6
Married to relatives other than first paternal/maternal cousins	471	18.5	842	21.8	1300	26.4	953	22.3
**Reproductive health and fertility behavior**								
*Age at first birth*								
< 20 years	2390	58.8	3072	54.1	3685	49.5	3076	45.8
20–34 years	1648	40.6	2588	45.6	3733	50.1	3593	53.5
35–49 years	23	0.6	17	0.3	28	0.4	42	0.6
*Gravidity*								
1–2	1306	32.2	2071	36.5	2980	40.0	2829	42.2
3–5	1808	44.5	2456	43.3	3233	43.4	3009	44.8
6 and above	947	23.3	1150	20.3	1233	16.6	873	13.0
*Parity*								
1–2 children	1245	30.7	2000	35.2	2885	38.7	2749	41.0
3–4 children	1142	28.1	1648	29.0	2249	30.2	2183	32.5
5 children and above	1674	41.2	2029	35.7	2312	31.1	1780	26.5
*Number of living children*								
None	71	1.7	100	1.8	80	1.1	83	1.2
1–2	1360	33.5	2138	37.7	3149	42.3	2944	43.9
3–4	1294	31.9	1737	30.6	2306	31.0	2222	33.1
5 and above	1336	32.9	1702	30.0	1912	25.7	1463	21.8
Ever terminated pregnancy*								
Yes	–	–	1352	23.8	2512	33.7	2166	32.3
No	–	–	4320	76.2	4935	66.3	4545	67.7
*Visits for antenatal care*								
Less than 4 visits	3410	85.8	3987	71.2	4713	63.4	2414	41.1
At least 4 visits	564	14.2	1611	28.8	2723	36.6	3452	58.9
*Deliveries by skilled birth attendants*								
Yes	743	18.5	2365	41.9	4112	55.2	4833	72.0
No	3285	81.5	3280	58.1	3312	44.5	1879	28.0
*Ideal family size*								
1–2	219	5.4	735	12.9	1055	14.2	1174	17.5
3–4	1045	25.8	3032	53.4	4200	56.4	3479	51.8
5 and above	2791	68.8	1910	33.6	2191	29.4	2059	30.7
*Desire for more children/Fertility intention*								
Wants more children	1748	43.7	2647	46.7	3592	49.0	3115	47.2
Wants no more	2254	56.3	3023	53.3	3745	51.0	3480	52.8
*Current use of contraception*								
Yes	475	11.7	1670	29.4	2774	37.3	2421	36.1
No	3586	88.3	4007	70.6	4672	62.7	4290	63.9

*Missing information indicates the non-availability of data within the respective PDHS wave

### Reproductive health and fertility behavior related characteristics

Table 2 demonstrates the characteristics related to women’s reproductive health and fertility behavior from 1990 to 2018. Results indicated that the majority of respondents gave their first birth at an age below 20 years and had multi-gravida pregnancies between 3 and 5 times (44.5%, 43.3%, 43.4%, and 44.8%). Parity of 5 children and more decreased over time (41.2%, 35.7%, 31.1%, and 26.5%). The proportion of deliveries conducted by unskilled birth attendants during the last pregnancy constantly decreased from 81.5% in 1990–1991 to 28.0% in 2017–2018. Findings also revealed the gradual improvement in women’s reproductive healthcare-seeking behaviors, particularly in availing at least 4 ANC visits and deliveries conducted by SBAs from 1990 to 2018. Furthermore, regarding women’s fertility behavior, the analysis indicated that most of the women (68.8%) reported an ideal family size of 5 children and above in 1990, which decreased over time to 3–4 children in 2017–2018 (51.8%). Although about half of the respondents showed no more desire for children or fertility intention in all four waves, there was a high—but overall decreasing—rate of not using contraception (88.3%, 70.6%, 62.7%, and 63.9%).

### Bivariate association of consanguinity with various factors

The association of consanguineous marriages with various factors—including sociodemographic characteristics, and reproductive health and fertility behaviors—amongst respondents of reproductive age in each of the four PDHS waves is presented in Table 3. Findings demonstrate the significant relationship of consanguinity with regions/provinces, urban/rural geographical classification, respondents’ education, wealth quintile, mass media exposure, and healthcare decision-making autonomy in all waves. Respondents’ age, employment status, and their husbands’ educational status were also found statistically significant in most of the PDHS waves. Furthermore, results highlight the significant relationship of consanguinity with respondents’ age at first birth, ANC visits, deliveries by SBAs, ideal family size, fertility intention, and current use of contraception in the majority of PDHS waves. In a few PDHS waves, a strong association of consanguineous unions was also observed with the number of living children, gravidity, and pregnancy termination.

**Table 3:** Relationship of consanguinity with sociodemographic characteristics, reproductive health and fertility behavior

Characteristics	PDHS (1990–1991)			PDHS (2006–2007)			PDHS (2012–2013)			PDHS (2017–2018)		
	n = 4061			n = 5677			n = 7446			n = 6711		
	Consan-guineous	Non-consan-guineous	*p*-value*	Consan-guineous	Non-consan-guineous	*p*-value*	Consan-guineous	Non-consan-guineous	*p*-value*	Consan-guineous	Non-consan-guineous	*p*-value*
**Sociodemographic characteristics and co-variates**												
*Regions/Provinces*												
Punjab	65.6	34.4	** < 0.01**	68.4	31.6	** < 0.01**	65.6	34.4	** < 0.01**	60	40	** < 0.01**
Sindh	61.6	38.4		73.0	27.0		71.4	28.6		73.8	26.2	
Baluchistan	70.3	29.7		72.3	27.7		69.7	30.3		64.7	35.3	
Khyber Pakhtunkhwa	51.9	48.1		56.1	43.9		60.3	39.7		61.7	38.3	
Gilgit Baltistan	–	–		–	–		51.8	48.2		–	–	
Islamabad	–	–		–	–		58.1	41.9		55.6	44.4	
FATA	–	–		–	–		–	–		56.4	43.6	
*Geographical classification*												
Urban	53.1	46.9	** < 0.01**	60.0	40.0	** < 0.01**	54.8	45.2	**﻿0.01**	58.6	41.4	** < 0.01**
Rural	67.0	33.0		71.4	28.6		71.1	28.9		66.2	33.8	
*Respondents’ age*												
15–24 years	67.3	32.7	** < 0.01**	70.1	29.9	0.12	70.4	29.6	** < 0.01**	68.8	31.2	** < 0.01**
25–34 years	62.4	37.6		66.9	33.1		64.8	35.2		62.1	37.9	
35 years and above	59.8	40.2		68.1	31.9		65.0	35.0		62.0	38.0	
*Respondents’ education status*												
Uneducated	65.0	35.0	** < 0.01**	70.5	29.5	** < 0.01**	71.4	28.6	** < 0.01**	69	31.0	** < 0.01**
Primary	64.8	35.2		67.5	32.5		66.8	33.2		65.3	34.7	
Secondary	48.2	51.8		62.3	37.7		58.2	41.8		59.7	40.3	
Higher	41.3	58.7		55.0	45.0		49.2	50.8		49.1	50.9	
*Husbands’ education status*												
Uneducated	61.1	38.9	** < 0.01**	68.7	31.3	0.45	67.6	32.4	** < 0.01**	66.8	33.2	** < 0.01**
Primary	68.9	31.1		69.2	30.8		69.8	30.2		69.6	30.4	
Secondary	63.5	36.5		67.2	32.8		65.4	34.6		62.7	37.3	
Higher	54.7	45.3		66.3	33.7		61.6	38.4		57.2	42.8	
*Respondents’ employment status*												
Unemployed	62.2	37.8	**0.03**	66.2	33.8	** < 0.01**	63.1	36.9	** < 0.01**	63.3	36.7	0.18
Employed	66.8	33.2		72.3	27.7		74.1	25.9		65.3	34.7	
*Husbands’ employment status*												
Unemployed	66.7	33.3	0.47	68.4	31.6	0.89	70.7	29.3	0.28	71.5	28.5	**0.03**
Employed	62.7	37.3		67.9	32.1		66.1	33.9		63.7	36.3	
*Mass media exposure*												
Yes	59.0	41.0	** < 0.01**	–	–	﻿–	64.0	36	** < 0.01**	61.6	38.4	** < 0.01**
No	66.0	34.0		–	–		71.4	28.6		67.1	32.9	
*Wealth quintile*												
Richest	59.9	40.1	** < 0.01**	61.3	38.7	** < 0.01**	54.7	45.3	** < 0.01**	51.4	48.6	** < 0.01**
Richer	60.1	39.9		62.7	37.3		56.6	43.4		58.8	41.2	
Middle	61.1	38.9		66.2	33.8		66.1	33.9		65.9	34.1	
Poorer	69.8	30.2		69.8	30.2		71.5	28.5		65.4	34.6	
Poorest	67.1	32.9		77.3	22.7		78.3	21.7		74.9	25.1	
*Respondent’s healthcare decision-making autonomy*												
Yes	–	–	–	–	–	–	63.2	36.8	** < 0.01**	62.6	37.4	**0.04**
No	–	–		–	–		69.2	30.8		65.1	34.9	
Reproductive health and fertility behaviors												
*Respondent’s age at first birth*												
< 20 years	63.9	36.1	0.32	69.5	30.5	**0.01**	71.3	28.7	**0.01**	68.0	32.0	**0.01**
20–34 years	61.8	38.2		66.3	33.7		61.1	38.9		60.1	39.9	
35–49 years	56.5	43.5		47.1	52.9		64.3	35.7		48.8	51.2	
*Gravidity*												
1–2	66.6	33.4	**0.01**	68.8	31.2	0.58	65.9	34.1	0.65	63.9	36.1	0.78
3–5	62.0	38.0		67.4	32.6		66.0	34.0		63.2	36.8	
6 and above	59.7	40.3		67.7	32.3		67.3	32.7		64.1	35.9	
*Parity*												
1–2 children	65.3	34.7	0.12	67.7	32.3	0.14	64.7	35.3	0.08	63.8	36.2	0.52
3–4 children	61.8	38.2		66.4	33.6		66.5	33.5		62.8	37.2	
5 children and above	62.1	37.9		69.4	30.6		67.7	32.3		64.5	35.5	
*Number of living children*												
None	70.4	29.6	** < 0.01**	75.8	24.2	0.40	75.0	25.0	0.25	75.6	24.4	0.07
1–2	66.3	33.7		67.9	32.1		65.4	34.6		63.8	36.2	
3–4	62.3	37.7		67.5	32.5		66.3	33.7		62.4	37.6	
5 and above	59.8	40.2		67.9	32.1		66.9	33.1		64.5	35.5	
*Ever terminated pregnancy*												
Yes	–	–	–	69.8	30.2	0.08**0.08**	70.1	29.9	** < 0.01**	64.3	35.7	0.42
No	–	–		67.3	32.7	﻿0.08	64.2	35.8		63.3	36.7	
*Visits for antenatal care*												
Less than 4 visits	63.6	36.4	**0.01**	64.7	35.3	** < 0.01**	69.9	30.1	** < 0.01**	66.9	33.1	** < 0.01**
At least 4 visits	58.1	41.9		69.3	30.7		59.7	40.3		60.1	39.9	
*Deliveries by skilled birth attendants*												
Yes	54.7	45.3	** < 0.01**	64.2	35.8	** < 0.01**	63.5	36.5	** < 0.01**	61.9	38.1	** < 0.01**
No	64.8	35.2		70.7	29.3		69.7	30.3		68.1	31.9	
*Ideal family size*												
1–2	49.5	50.5	** < 0.01**	60.8	39.2	** < 0.01**	57.3	42.7	** < 0.01**	56.9	43.1	** < 0.01**
3–4	60.2	39.8		67.7	32.3		64.3	35.7		62.9	37.1	
5 and above	65.0	35.0		71.1	28.9		74.1	25.9		68.6	31.4	
*Desire for more children/Fertility intention*												
Wants more children	69.2	30.8	** < 0.01**	68.9	31.1	0.12	68.1	31.9	** < 0.01**	65.6	34.4	**0.01**
Wants no more	58.2	41.8		67.0	33.0		64.6	35.4		62.4	37.6	
*Current use of contraception*												
Yes	51.9	48.1	** < 0.01**	65.8	34.2	**0.03**	63.6	36.4	** < 0.01**	59.5	40.5	** < 0.01**
No	64.4	35.6		68.8	31.2		67.7	32.3		66.0	34.0	

*P-value is based on the Chi-square test. All significant values (*p* < 0.05) are marked in bold

### Bivariable and multivariable logistic regression

Table 4 depicts the results of the bivariable logistic regression analysis of consanguinity with respondents’ sociodemographic characteristics, reproductive health, and fertility behaviors. Almost all sociodemographic variables in the four PDHS waves were significantly associated with consanguinity—at least one category per item. However, the husband’s employment status was only significant for 2017–2018. Furthermore, gravidity and parity, and the number of children showed nearly no significant associations.

**Table 4:** Bivariable logistic regression of Consanguinity with sociodemographic characteristics, reproductive health and fertility behavior

Characteristics	PDHS (1990–1991)			PDHS (2006–2007)			PDHS (2012–2013)			PDHS (2017–2018)		
	n = 4061			n = 5677			n = 7446			n = 6711		
	OR	95% CI	*p*-value	OR	95% CI	*p*-value	OR	95% CI	*p*-value	OR	95% CI	*p*-value
**Sociodemographic characteristics and co-variates**												
*Regions/Provinces*												
Khyber Pakhtunkhwa	1			1			1			1		
Punjab	1.76	1.47–2.12	** < 0.01**	1.70	1.45–1.99	** < 0.01**	1.25	1.09–1.43	** < 0.01**	0.93	0.81–1.07	0.30
Sindh	1.48	1.20–1.84	** < 0.01**	2.11	1.76–2.53	** < 0.01**	1.64	1.39–1.92	** < 0.01**	1.74	1.47–2.05	** < 0.01**
Baluchistan	2.19	1.50–3.19	** < 0.01**	2.05	1.51–2.78	** < 0.01**	1.51	1.17–1.95	** < 0.01**	1.13	0.89–1.45	0.31
Gilgit Baltistan	–	–	–	–	–	–	0.70	0.41–1.21	0.20	–	–	–
Islamabad	–	–	–	–	–	–	0.88	0.43–1.82	0.75	0.76	0.44–1.32	0.34
FATA	–	–	–	–	–	–				0.79	0.56–1.12	0.19
*Geographical classification*												
Rural	1			1			1			1		
Urban	0.55	0.48–0.64	** < 0.01**	0.60	0.53–0.68	** < 0.01**	0.49	0.45–0.55	** < 0.01**	0.72	0.65–0.80	** < 0.01**
*Respondents’ age*												
15–24 years	1			1			1			1		
25–34 years	0.80	0.68–0.94	** < 0.01**	0.86	0.75–0.99	**0.04**	0.77	0.68–0.87	** < 0.01**	0.75	0.65–0.84	** < 0.01**
35 years and above	0.72	0.60–0.86	** < 0.01**	0.91	0.77–1.07	0.27	0.78	0.67–0.90	** < 0.01**	0.74	0.63–0.86	** < 0.01**
*Respondents’ education status*												
Uneducated	1			1			1			1		
Primary	0.99	0.79–1.24	0.93	0.87	0.74–1.02	0.08	0.81	0.70–0.92	** < 0.01**	0.84	0.73–0.97	**0.02**
Secondary	0.50	0.41–0.61	** < 0.01**	0.69	0.59–0.81	** < 0.01**	0.55	0.49–0.63	** < 0.01**	0.66	0.58–0.75	** < 0.01**
Higher	0.37	0.20–0.67	** < 0.01**	0.51	0.41–0.64	** < 0.01**	0.38	0.33–0.45	** < 0.01**	0.43	0.37–0.50	** < 0.01**
*Husbands’ education status*												
Uneducated	1			1			1			1		
Primary	1.41	1.17–1.69	** < 0.01**	1.02	0.86–1.21	0.79	1.11	0.95–1.28	0.17	1.13	0.96–1.33	0.12
Secondary	1.11	0.96–1.29	0.16	0.94	0.82–1.07	0.33	0.91	0.81–1.02	0.10	0.83	0.73–0.94	**0.01**
Higher	0.77	0.56–1.05	0.09	0.89	0.75–1.06	0.21	0.77	0.66–0.89	** < 0.01**	0.66	0.57–0.77	** < 0.01**
*Respondents’ employment status*												
Unemployed	1			1			1			1		
Employed	1.22	1.02–1.45	**0.02**	1.33	1.17–1.51	** < 0.01**	1.67	1.49–1.87	** < 0.01**	1.09	0.95–1.24	0.19
*Husbands’ employment status*												
Unemployed	1			1			1			1		
Employed	0.84	0.52–1.35	0.47	0.97	0.71–1.35	0.89	0.79	0.54–1.18	0.25	0.70	0.50–0.98	**0.03**
*Mass media exposure*												
No	1						1			1		
Yes	0.74	0.65–0.85	** < 0.01**	–	–	–	0.71	0.64–0.79	** < 0.01**	0.78	0.71–0.87	** < 0.01**
*Respondents’ healthcare decision-making autonomy*												
No							1			1		
Yes	–	–	–	–	–	–	0.76	0.69–0.84	** < 0.01**	0.89	0.83–0.96	** < 0.01**
*Wealth quintile*												
Poorest	1			1			1			1		
Poorer	1.13	0.89–1.43	0.31	0.68	0.57–0.81	** < 0.01**	0.69	0.59–0.82	** < 0.01**	0.63	0.54–0.74	** < 0.01**
Middle	0.77	0.61–0.96	**0.02**	0.57	0.48–0.69	** < 0.01**	0.54	0.46–0.63	** < 0.01**	0.65	0.55–0.76	** < 0.01**
Richer	0.74	0.60–0.92	** < 0.01**	0.49	0.41–0.59	** < 0.01**	0.36	0.31–0.42	** < 0.01**	0.47	0.41–0.56	** < 0.01**
Richest	0.73	0.59–0.90	** < 0.01**	0.46	0.39–0.56	** < 0.01**	0.33	0.28–0.39	** < 0.01**	0.35	0.30–0.41	** < 0.01**
**Reproductive health and fertility behaviors**												
*Respondents’ age at first birth*												
< 20 years	1			1			1			1		
20–34 years	0.91	0.80–1.04	0.17	0.86	0.77–0.96	**0.01**	0.63	0.57–0.70	** < 0.01**	0.71	0.64–0.78	** < 0.01**
35–49 years	0.69	0.30–1.58	0.38	0.37	0.14–0.97	**0.04**	0.75	0.34–1.65	0.48	0.45	0.24–0.83	**0.01**
*Gravidity*												
6 and above	1			1			1			1		
3–5	1.10	0.94–1.29	0.23	0.98	0.85–1.14	0.86	0.94	0.81–1.07	0.37	0.96	0.82–1.12	0.61
1–2	1.35	1.13–1.60	** < 0.01**	1.05	0.90–1.23	0.51	0.94	0.82–1.08	0.41	0.99	0.84–1.16	0.91
*Parity*												
5 children and above	1			1			1			1		
3–4 children	0.98	0.84–1.15	0.88	0.87	0.76–1.00	**0.05**	0.95	0.84–1.07	0.38	0.93	0.81–1.06	0.26
1–2 children	1.15	0.98–1.34	0.07	0.92	0.81–1.05	0.23	0.88	0.78–0.98	**0.02**	0.97	0.86–1.10	0.65
*Number of living children*												
5 children and above	1			1			1			1		
3–4 children	1.11	0.94–1.29	0.19	0.98	0.85–1.13	0.79	0.97	0.85–1.11	0.69	0.91	0.79–1.05	0.20
1–2 children	1.32	1.13–1.54	** < 0.01**	1.00	0.87–1.14	0.98	0.93	0.83–1.05	0.28	0.97	0.85–1.11	0.68
None	1.61	0.95–2.71	0.07	1.45	0.91–2.32	0.12	1.44	0.86–2.41	0.16	1.70	1.02–2.84	**0.04**
*Ever terminated pregnancy*												
No				1			1			1		
Yes	–	**–**	**–**	2.31	2.06–2.59	** < 0.01**	1.31	1.18–1.45	** < 0.01**	1.10	1.02–1.19	**0.01**
*Visits for antenatal care*												
Less than 4 visits	1			1			1			1		
At least 4 visits	0.79	0.66–0.95	**0.01**	1.83	1.65–2.03	** < 0.01**	0.64	0.58–0.70	** < 0.01**	0.74	0.67–0.83	** < 0.01**
*Deliveries by skilled birth attendants*												
No	1			1			1			1		
Yes	0.66	0.56–0.77	** < 0.01**	1.79	1.64–1.95	** < 0.01**	0.75	0.68–0.83	** < 0.01**	0.76	0.68–0.85	** < 0.01**
*Ideal family size*												
5 children or above	1			1			1			1		
3–4 children	0.81	0.70–0.94	**0.01**	0.63	0.53–0.75	** < 0.01**	0.62	0.56–0.70	** < 0.01**	0.78	0.69–0.87	** < 0.01**
1–2 children	0.53	0.40–0.69	** < 0.01**	0.85	0.75–0.96	**0.01**	0.46	0.40–0.54	** < 0.01**	0.60	0.52–0.70	** < 0.01**
*Desire for more children/Fertility intention*												
Wants more children	1			1			1			1		
Wants no more	0.62	0.54–0.71	** < 0.01**	0.92	0.82–1.02	0.12	0.86	0.78–0.94	** < 0.01**	0.87	0.78–0.96	**0.01**
*Current use of contraception*												
No	1			1			1			1		
Yes	0.59	0.49–0.72	** < 0.01**	0.87	0.77–0.98	**0.02**	0.84	0.76–0.92	** < 0.01**	0.76	0.68–0.84	** < 0.01**

All significant values (*p* < 0.05) are marked in bold

When interpreting the results of the multivariable logistic regression analysis (Table 5), one needs to keep in mind that not all variables and categories have been assessed in all four waves. However, the results highlight that respondent’s age was not significantly associated with consanguinity. The region and geographical classification were significant predictors of consanguinity, except for 2017–2018. The impact of education was contrary between women and their husbands: Women with higher education had a lower likelihood for consanguineous marriages, whereas men with higher education were more likely to marry their relatives. The strength of the association for both variables reduced over time. Employment status both for women and their husbands showed inconclusive results—the only significant association was found for employed women showing a higher likelihood of consanguinity in 2012–2013 (AOR = 1.23, 95% CI 1.08–1.39, *p* < 0.01).

**Table 5:** Bivariable logistic regression of consanguinity with sociodemographic characteristics, reproductive health and fertility behaviour

Characteristics	PDHS (1990–1991)			PDHS (2006–2007)			PDHS (2012–2013)			PDHS (2017–2018)		
	n = 4061			n = 5677			n = 7446			n = 6711		
	AOR	95% CI	*p*-value	AOR	95% CI	*p*-value	AOR	95% CI	*p*-value	AOR	95% CI	*p*-value
**Sociodemographic characteristics and co-variates**												
*Regions/Provinces*												
Khyber Pakhtunkhwa	1			1			1			1		
Punjab	1.88	1.53–2.30	**< 0.01**	1.92	1.62–2.28	**< 0.01**	1.64	1.39–1.92	**< 0.01**	1.19	1.00–1.42	**0.04**
Sindh	1.81	1.42–2.31	**< 0.01**	2.36	1.93–2.88	**< 0.01**	1.91	1.58–2.29	**< 0.01**	1.96	1.59–2.41	**< 0.01**
Baluchistan	2.08	1.36–3.18	**< 0.01**	1.85	1.34–2.56	**< 0.01**	1.22	0.92–1.62	0.16	0.93	0.68–1.29	0.68
Gilgit Baltistan	–	–	–	–	–	–	0.57	0.32–1.01	**0.05**		–	–
Islamabad	–	–	–	–	–	–	1.63	0.76–3.46	0.21	1.32	0.72–2.39	0.36
FATA	–	–	–	–	–	–				0.56	0.37–0.85	**< 0.01**
*Geographical classification*												
Rural	1			1			1			1		
Urban	0.63	0.52–0.76	**< 0.01**	0.66	0.57–0.77	**< 0.01**	0.74	0.64–0.85	**< 0.01**	1.09	0.95–1.26	0.20
*Respondents’ age*												
15–24 years	1			1			1			1		
25–34 years	0.89	0.72–1.24	0.35	0.95	0.79–1.14	0.58	0.92	0.79–1.08	0.34	0.86	0.73–1.02	0.09
35 years and above	0.83	0.60–1.14	0.25	1.01	0.78–1.29	0.94	0.93	0.74–1.17	0.54	0.84	0.66–1.06	0.16
*Respondents’ education status*												
Uneducated	1			1			1			1		
Primary	0.97	0.74–1.25	0.79	0.92	0.77–1.09	0.34	1.01	0.86–1.19	0.86	0.89	0.76–1.07	0.23
Secondary	0.52	0.39–0.69	**< 0.01**	0.78	0.64–0.95	**0.01**	0.83	0.69–0.98	**0.02**	0.82	0.69–0.97	**0.02**
Higher	0.35	0.17–0.73	**< 0.01**	0.58	0.43–0.79	**< 0.01**	0.59	0.46–0.75	**< 0.01**	0.66	0.53–0.83	**< 0.01**
*Husbands’ education status*												
Uneducated	1			1			1			1		
Primary	1.54	1.26–1.87	**< 0.01**	1.14	0.96–1.37	0.14	1.29	1.10–1.53	**< 0.01**	1.39	1.15–1.67	**< 0.01**
Secondary	1.64	1.36–1.97	**< 0.01**	1.30	1.12–1.52	**< 0.01**	1.46	1.27–1.68	**< 0.01**	1.35	1.14–1.59	**0.01**
Higher	2.12	1.42–3.17	**< 0.01**	1.52	1.22–1.90	**< 0.01**	1.77	1.46–2.13	**< 0.01**	1.27	1.04–1.55	**0.02**
*Respondents’ employment status*												
Unemployed	1			1			1			1		
Employed	1.07	0.88–1.30	0.47	1.04	0.91–1.20	0.54	1.23	1.08–1.39	**< 0.01**	0.90	0.77–1.06	0.22
*Husbands’ employment status*												
Unemployed	1			1			1			1		
Employed	0.76	0.46–1.27	0.29	0.92	0.65–1.29	0.62	0.72	0.47–1.09	0.12	0.69	0.46–1.02	0.06
*Mass media exposure*												
No	1						1			1		
Yes	0.91	0.77–1.08	0.28	–	–	–	1.01	0.88–1.16	0.85	1.04	0.90–1.20	0.58
*Respondents’ healthcare decision-making autonomy*												
No							1			1		
Yes	–	–	–	–	–	–	0.83	0.74–0.92	**< 0.01**	0.92	0.82–1.04	0.17
*Wealth quintile*												
Poorest	1			1			1			1		
Poorer	1.08	0.84–1.40	0.52	0.78	0.64–0.94	**0.01**	0.79	0.66–0.95	**0.01**	0.67	0.54–0.83	**< 0.01**
Middle	0.85	0.66–1.08	0.18	0.67	0.55–0.82	**< 0.01**	0.63	0.52–0.77	**< 0.01**	0.72	0.57–0.90	**< 0.01**
Richer	0.82	0.65–1.04	0.10	0.62	0.49–0.78	**< 0.01**	0.45	0.36–0.55	**< 0.01**	0.55	0.43–0.71	**< 0.01**
Richest	0.89	0.71–1.13	0.34	0.73	0.56–0.96	**0.02**	0.53	0.41–0.69	**< 0.01**	0.47	0.35–0.62	**< 0.01**
**Reproductive health and fertility behaviors**												
*Respondents’ age at first birth *												
**< **20 years	1			1			1			1		
20–34 years	0.98	0.83–1.16	0.87	0.94	0.82–1.08	0.39	0.73	0.65–0.83	**< 0.01**	0.82	0.72–0.95	**< 0.01**
35–49 years	0.99	0.38–2.60	0.98	0.30	0.11–0.83	**0.02**	0.86	0.37–1.99	0.72	0.83	0.39–1.76	0.62
*Gravidity*												
6 and above	1			1			1			1		
3–5	0.88	0.67–1.15	0.35	1.08	0.86–1.37	0.48	1.15	0.93–1.43	0.20	1.21	0.93–1.56	0.15
1–2	0.87	0.52–1.46	0.61	1.52	1.01–2.31	**0.05**	1.60	1.11–2.32	**0.01**	1.10	0.72–1.70	0.66
*Parity*												
5 children and above	1			1			1			1		
3–4 children	0.73	0.54–0.97	**0.03**	0.64	0.48–0.86	**< 0.01**	1.00	0.78–1.29	0.97	1.16	0.87–1.54	0.32
1–2 children	0.46	0.28–0.75	**< 0.01**	0.58	0.37–0.89	**0.01**	0.79	0.55–1.15	0.23	1.08	0.72–1.63	0.70
*Number of living children*												
5 children and above	1			1			1			1		
3–4 children	1.37	0.98–1.91	0.06	1.53	1.10–2.13	**0.01**	1.23	0.93–1.64	0.15	0.91	0.65–1.26	0.58
1–2 children	2.48	1.34–4.58	**< 0.01**	1.43	0.84–2.44	0.19	1.23	0.78–1.94	0.36	1.11	0.66–1.88	0.69
None	2.83	1.16–6.88	**0.02**	2.15	1.02–4.54	**0.04**	1.35	0.66–2.77	0.41	1.57	0.72–3.44	0.26
*Ever terminated pregnancy *												
No				1			1			1		
Yes	**–**	**–**	**–**	1.10	0.96–1.27	0.16	1.29	1.15–1.44	**< 0.01**	1.11	0.99–1.26	0.08
*Visits for antenatal care *												
Less than 4 visits	1			1			1			1		
At least 4 visits	1.45	1.13–1.86	**< 0.01**	1.00	0.86–1.16	0.97	0.85	0.75–0.97	**0.02**	0.99	0.87–1.13	0.94
*Deliveries by skilled birth attendants*												
No	1			1			1			1		
Yes	0.84	0.67–1.04	0.11	0.91	0.79–1.04	0.18	1.08	0.96–1.22	0.22	0.93	0.80–1.08	0.35
*Ideal family size*												
5 children and above	1			1			1			1		
3–4 children	1.52	1.10–2.09	**0.01**	0.98	0.84–1.13	0.74	0.69	0.61–0.80	**< 0.01**	0.87	0.59–0.87	0.06
1–2 children	1.55	1.13–2.14	**0.01**	0.74	0.60–0.92	**< 0.01**	0.64	0.53–0.78	**< 0.01**	0.72	0.74–1.01	**< 0.01**
*Desire for more children/Fertility intention*												
Wants more children	1			1			1			1		
Wants no more	1.28	1.08–1.52	**< 0.01**	1.03	0.88–1.20	0.69	1.03	0.89–1.18	0.66	0.90	0.78–1.04	0.16
*Current use of contraception*												
No	1			1			1			1		
Yes	0.84	0.67–1.05	0.13	1.04	0.91–1.19	0.59	1.06	0.95–1.19	0.29	0.90	0.81–1.04	0.17

All significant values (*p* < 0.05) are marked in bold

All significant values (*p* < 0.05) are marked in bold

In 1990–1991, the wealth index was not significantly associated with consanguinity, but for all other waves, a higher wealth quintile was linked with a lower likelihood of consanguineous marriages. For example, in 2012–2013 (AOR = 0.53, 95% CI 0.41–0.69, *p* < 0.01) and 2017–2018 (AOR = 0.47, 95% CI 0.35–0.62, *p* < 0.01) the likelihood was halved in the richest wealth quintile compared to the poorest one. There was no impact of mass media exposure and respondents’ healthcare decision-making autonomy, except for 2012–2013, where women having decision-making autonomy were less likely to be married to relatives (AOR = 0.83, 95% CI 0.74–0.92, *p* < 0.01).

As already shown in the bivariable logistic regression models, respondents’ age at first birth, gravidity and parity, and the number of children showed almost no or only in some years or categories significant associations. Nevertheless, there is a trend younger age at first birth and lower gravidity is linked with consanguineous marriages. In 1990–1991 and 2006–2007, a lower number of children was associated with a higher likelihood for consanguineous marriages, ever having terminated pregnancy was only significantly associated with a higher likelihood for consanguinity in 2012–2013 (AOR = 1.29, 95% CI 1.15–1.44, *p* < 0.01). Visits of ANC and deliveries by SBAs were also no significant predictors, except for single exceptions. However, an ideal family size of less than five children was almost entirely significantly associated with not marrying a relative. The use of contraceptives was not significantly associated with consanguineous marriages.

## Discussion

With the advanced research and expansion of knowledge in public health and social sciences, the topic of consanguineous unions has received higher importance. From the beginning of mankind, consanguinity or close-kin marriages were socially and culturally deeply rooted. Although it is presumed that the rates of consanguineous marriages decline with modernization and literacy, this is not transferable to all countries [30]. Presently, consanguinity is widely popular and respected in many communities, particularly in Muslims [2, 3]. Pakistan ranks amongst those countries, where the highest prevalence of consanguinity is still in vogue [3, 22, 25]. This article examined the trends of consanguineous marriages over approximately three decades, from 1990 to 2018, and their association with women’s reproductive health and fertility behavior in Pakistan. It is an effort to bridge the gap in existing literature, documenting the relevance of consanguinity with reproductive health and fertility behavior amongst women, who had given births in five years preceding each of the four PDHS waves.

The results showed a varied trend of consanguineous marriages in Pakistan, which increased from 63.0% in 1990–1991 to 67.9% in 2006–2007, however, declined to 66.2% during 2012–2013 and 63.6% in 2018. This highlights the fact that the popularity of consanguineous unions is not declining in the country, because of social, cultural, religious, and economic advantages, which outweigh the disadvantages given the population [31]. In particular, consanguinity promotes family stability, inheritance, and spouse compatibility, nonetheless lessens hidden financial risks [6, 7, 16, 32]. These results are similar to other studies, carried out in many subpopulations within Pakistan [33], such as northern Punjab [34, 35], southern Khyber Pakhtunkhwa [36], Balochistan [37], Kashmir [38], and also in other Arab countries [1, 39], Yemen [40], Qatar [41] and Algeria [42]. Contrary, these results are not consistent with some of the research, where a decreasing trend in consanguineous unions was reported over time [43, 44].

This research also reiterated that consanguinity is associated with sociodemographic characteristics, as results demonstrated that consanguineous marriages are more prevalent amongst uneducated women, living in rural areas, and with poorer wealth status. These findings are comparable to other studies, where less-educated women get married to their cousins at a younger age, particularly in poor traditional rural areas [10, 45–48]. This highlights the need to educate and empower young girls, enabling them to make better informed decisions for their reproductive life to ensure their well-being. Previous empirical results found a strong association of consanguineous unions with women’s reproductive health and fertility behaviors. Findings demonstrated that those women who married their cousins were more likely to give first birth at a younger age (between 20 and 34 years). Although not entirely significant in our analysis, we can also confirm this result. Our findings correspond to the previous studies, showing that consanguinity is associated with higher fertility rates and larger family sizes, which affects the health of both mothers and children [5, 8, 13, 22, 24, 25], particularly in the case of younger women [49]. Thus, there is a need to educate communities about linkages of consanguinity with poor reproductive health, adverse impact on fertility outcomes, and overall family health. This research emphasizes educating families regarding implications of consanguinity and associated health risks, through increasing public awareness, providing informational material, promoting health education, and enhancing capacities of primary healthcare and outreach workers to counsel communities effectively on health and social issues related to consanguineous marriages. It is pertinent to actively engage all key stakeholders in the public and private sector, particularly healthcare providers, outreach workers, and social mobilizers to elucidate the health and social effects of consanguineous marriages and promote healthy mothers, children, and communities.

### Limitations

Since this research analyzed the four waves of PDHS from 1990 to 2018, few variables were not uniform and found missing, particularly in PDHS 1990–1991 and 2006–2007, such as regions/provinces, mass media exposure, respondent’s healthcare decision-making autonomy, and pregnancy termination. Due to the cross-sectional design, we are not able to draw any causal conclusions. When interpreting the results, one needs to consider that some of the variables might be predictors of consanguineous marriages (such as low education), whereas others are effects (such as visits of ANC and deliveries by SBAs) or both (such as ideal family size).

## Conclusion

This research concludes that consanguineous marriages are predominant in Pakistan, particularly in the context of the large power structure and patriarchal society. Findings revealed that consanguinity is associated with sociodemographic characteristics and women’s reproductive health and fertility behaviors in Pakistan. The high prevalence of consanguineous marriages and their implications on women’s health is essential to be considered in health policies. Owing to dilute these prevailing socio-cultural practices, a nationwide public education program has to be conducted, engaging key stakeholders (including health managers, healthcare providers, and outreach workers) and highlighting the risk factors associated with consanguinity to minimize the adverse health outcomes. Further, there is also a dire need to actively engage public health and reproductive health professionals to promote the health and wellbeing of the female population. Healthcare providers, outreach workers, and social mobilizers may play a critical role in this regard, particularly in identifying the consanguineous couples within their serving community, counselling them and providing information on potential risk factors, and enabling them to make informed choices regarding their reproductive health. Appropriate counselling, health educational, and promotional programmes related to consanguinity should be designed and launched at health facilities and community level to build capacities of healthcare providers and raise awareness amongst the general population on danger signs. Though there are multiple socio-cultural and economic benefits of consanguinity perceived by women in Pakistan, improvements in health literacy and behavior change will endorse an attitudinal change in society.

## Data Availability

Data is available from the Demographic and Health Survey program: https://dhsprogram.com/.

## Abbreviations

ANC: Antenatal care
AOR: Adjusted odds ratio
CI: Confidence interval
OR: Odds ratio
PDHS: Pakistan Demographic and Health Survey
SBA: Skilled birth attendant
SPSS: Statistical Package for the Social Sciences
